# T cell redirection as a new standard of care for relapsed multiple myeloma: impact on inpatient capacity, financial burden and infrastructural requirements in Germany

**DOI:** 10.1038/s41409-024-02505-x

**Published:** 2025-01-22

**Authors:** P. Ahmadi, W. Alsdorf, L. Leypoldt, R. Kosch, C. Schaefers, N. Gagelmann, F. Ayuk, F. Kron, K. Weisel

**Affiliations:** 1 Controlling University Medical Center Hamburg-Eppendorf, Hamburg, Germany; 2 Department of Stem Cell Transplantation, University Medical Center Hamburg-Eppendorf, Hamburg, Germany; 3 Department of Oncology, Hematology and Bone Marrow Transplantation with Section Pneumology, University Cancer Center Hamburg-Eppendorf, Hamburg, Germany; 4 VITIS Healthcare Group, Cologne, Germany; 5 Department I of Internal Medicine, Faculty of Medicine and University Hospital Cologne, University of Cologne, Cologne, Germany; 6 Fachhochschule für Oekonomie & Management (FOM) University of Applied, Sciences, Essen, Germany

**Keywords:** Public health, Epidemiology

## To the Editor:

Multiple myeloma (MM) is a complex and heterogenous condition and is generally incurable. Although there has been an expansion in the number and range of treatments, nearly all patients eventually relapse. Several new innovative treatment approaches are under development to meet the clinical need for refractory patients [1]. Choosing the optimal treatment for every patient is challenging and depends on the right combination of agents and their mode of action, the stage of the disease, the patient’s comorbidities, the patient’s level of fitness, the availability of the therapy and the severity of side effects. For patients who have already undergone several prior treatment lines and developed multiple resistances, it is often difficult to achieve a deep and sustained response [2, 3]. During the past years new generation immunotherapeutics were developed leading to so far unprecedented outcomes in response and survival in refractory patients and found their way into standard of care approaches from first relapse. These T cell redirection strategies include CAR-T cell therapy with ciltacabtagene autoleucel (cilta-cel) and idecabtagene vicleucel (ide-cel) as well as the bispecific antibodies teclistamab, elranatamab, and soon expected linvoseltamab (all targeting B cell maturation antigen, BCMA) and talquetamab (targeting G protein coupled receptor class C group 5 member D, GPRC5D) in the approved setting, with even more under development. Especially their potential to improve outcomes and patient survival will lead to a broad application in earlier lines and in combination treatment approaches. These T cell redirection drugs differ in their manufacturing and availability. Some are off the shelf products like bispecific antibodies (bsAb) with a *de facto* immediate availability while autologous CAR-T cells require a complex and time consuming manufacturing process before reinfusion. Prior to CAR-T cell reinfusion, patients receive a lymphodepleting regimen. The selection of patients for treatment must be conducted with the utmost care and precision. Currently, the standard practice is to hospitalize patients for close monitoring of cytokine release syndrome (CRS) and immune effector cell-associated neurotoxicity syndrome (ICANS). Despite the efficacy of certain strategies in alleviating the side effects, CAR-T therapy is frequently employed in an inpatient setting for close monitoring. For the currently approved bsAbs, step-up dosing is needed when treatment is introduced which also requires an inpatient setting for 8-10 days according to the chosen construct due to the frequent occurrence of CRS during the first doses. Myeloma patients who used to be treated solely as outpatients are now becoming (temporary) inpatients and require additional hospital capacity at a time when hematological diseases in general are becoming more common in an aging population. In addition, high-dose therapy with autologous stem cell transplantation continues to be performed in an inpatient setting as first line treatment. Should CAR-T therapy now be employed in the second line, hospitals will be required to provide additional inpatient capacity to ensure the adequate treatment for these patients. For further treatment lines after CAR-T therapy, additional hospitalizations are needed for safe step-up dosing of the respective T cell engagers. In this study, we analyzed current trends and calculated the nationwide financial impact of the administration of cilta-cel for the treatment of MM based on experiences of our tertiary care center (Table 1).

**Table 1:** Median prior lines of treatment without bridging (min-max)/n = number of patients.

Year	Cilta-cel	Ide-cel
2022	–/(*n* = 0)	4 (2–8)/(*n* = 20)
2023	3 (2–4)/(*n* = 5)	4 (2–10)/(*n* = 11)
2024 (* as of 30.06.2024)	3 (1–6)/(*n* = 14)	8 (8–8)/(*n* = 1)
Total	3 (1–6)/(*n* = 19)	4 (2–10)/(*n* = 32)

On the German market, two BCMA-specific CAR-T-cell therapies from two manufacturers are currently approved for patients diagnosed with relapsed and refractory multiple myeloma (RRMM) from first relapse on. Ide-cel is currently indicated for the treatment of relapsed and refractory multiple myeloma in adult patients who have received at least two prior therapies, including an immunomodulator, a proteasome inhibitor and an anti-CD38 antibody, and have shown disease progression during the last therapy [4]. Clinical efficacy and safety was demonstrated in the KarMMa trials [5–8]. Cilta-cel is currently indicated for the treatment of adult patients with RRMM who have previously received at least one prior therapy, including an immunomodulator and a proteasome inhibitor, and who have shown disease progression during the last therapy and are refractory to lenalidomide. Prior to infusion with CAR-T cells, bridging therapy should be considered in order to reduce the tumor burden or stabilize the disease. Three days of chemotherapy are given to deplete lymphocytes and facilitate CAR-T-expansion. According to the cilta-cel and ide-cel prescribing information, the infusion of T cells should take place 5 to 7 (cilta-cel) and 2 to 9 days with 2 days pause (ide-cel) after start of lymphodepletion. Patients should be monitored daily at a qualified clinical facility for 10 days (ide-cel) and 14 days (cilta-cel) following CAR-T infusion for signs and symptoms of CRS, neurologic events, and other toxicities. If the required days of lymphodepletion, recovery and monitoring are added together, this results in an inpatient stay of 21 days for cilta-cel [9] and 16 days for ide-cel [4]. Our experience to date shows that inpatient monitoring after CAR-T therapy for myeloma leads to excellent safety and that toxicity can thus be controlled very well. The impact of these treatments on inpatient capacities will be outlined based on data from our tertiary academic center. In 2022–2024 (as of 30.06.2024), 51 patients were treated for RRMM with the commercial CAR-T cell products ide-cel and cilta-cel. The indication for CAR-T was made after a median of 4 lines and the patients received the CAR-T cells after a median of 5 lines.

For 2024, 28 inpatients are estimated to be treated with cilta-cel alone. By extending the indication to patients who had received only one previous line of therapy, we assume a total number of 52 per year for cilta-cel. The median length of stay for these patients is currently 22 days, ranging from 18 to 32 days and thus corresponds to the prescribing information of cilta-cel. This corresponds to a bed capacity of 28 inpatients × 22 days = 616 inpatient days and equates to around 1.69 beds (normal ward and intensive care unit combined) with a bed occupancy of 100%. With a total increase to 52 cases, the need for inpatient beds rises to 3.13 beds and describes an additional need of 1.44 beds per year for this therapy indication. The NUB (“Neue Untersuchungs- und Behandlungsmethoden”) fee for cilta-cel is currently € 285,000, which corresponds to the purchase price of the cells alone. In addition, there are the costs for inpatient treatment and care. The diagnosis related group (DRG) remuneration, including the remuneration for inpatient care costs, averages € 14,981 per case and is mainly attributable to DRG R61G and R61H. With 52 inpatient cases per year, the total cost for the CAR-T cell product cilta-cel alone will be € 14.8 million for our treatment center. In a nationwide consideration of Germany, the question arises as to how many patients in statutory health insurance belong to the target population and what bed requirement results from this. Referring to several dossiers from the “Gemeinsamer Bundesausschuss” regarding the “Nutzenbewertung” (health technology assessment) of various drugs for the treatment of multiple myeloma [10], the number is estimated to be between 3000–4000 patients/year. This results in additional 180 beds (3000 inpatients × 22 days / 365 days per year) and costs for CAR-T amounting to €855 million to €1.14 billion, assuming cilta-cel for therapy. Additionally, there are costs for the DRG amounting to €44.9 million to €59.9 million.

In conclusion, the advent of novel generation immunotherapeutics in the treatment of multiple myeloma has resulted in a notable surge in the demand for inpatient facilities to administer these medications to all eligible patients, ensuring their safety and the delivery of high-quality care through the provision of appropriate monitoring and the availability of a trained interprofessional team. It is imperative that adequate measures be implemented to ensure that the positive impact of these treatments on prognosis is translated in the real-world setting for patients with multiple myeloma. If we further consider the impact of inpatient bsAb step-up dosing on bed capacity, the enormous increase in demand for hospital beds becomes even clearer. Of note, with the integration of bsAbs into earlier treatment lines, further approval of additional bsAbs, and the possibility of receiving various immunotherapeutics over time (e.g., CAR-T [2nd line] followed by bsAbs [3rd line] followed by another bsAb [4th] etc.), it is anticipated that the demand for inpatient hospital beds will continue to increase even further and will accelerate the discussion about the ambulantization of monitoring after CAR-T therapy or bsAb. However, not all patients are hospitalized during bsAb step-up dosing and in a growing number of centers, patients are sent home with a caregiver with clear instructions for contacting the hospital in a case of adverse events. Potential strategies to address some of these challenges involve equipment to measure vital signs at home for patient monitoring [11] as well as prophylactic use of tocilizumab [12] to reduce inpatient days. There are also outpatient programs available in certain hospitals for prescribing bsAbs [13] and CAR-T therapies [14] to reduce the number of days patients spend in the hospital. While these measures will certainly alleviate some of the demand, there will still be an increasing need for inpatient capacities due to the novel generation immunotherapeutics.
